# Variations in the proliferative activity of the peripheral retina correlate with postnatal ocular growth in squamate reptiles

**DOI:** 10.1002/cne.24677

**Published:** 2019-03-28

**Authors:** Julia Eymann, Lotta Salomies, Simone Macrì, Nicolas Di‐Poï

**Affiliations:** ^1^ Program in Developmental Biology, Institute of Biotechnology University of Helsinki Helsinki Finland

**Keywords:** development, evolution, ocular growth, peripheral retina, progenitors, RRID: AB_143157, RRID: AB_143165, RRID: AB_1795608, RRID: AB_221448, RRID: AB_2239761, RRID: AB_2341193, RRID: AB_2734716, RRID: AB_305426, RRID: AB_305702, RRID: AB_314691, RRID: AB_561007, RRID: SCR_007353, RRID: SCR_014199, RRID: SCR_016299, squamates

## Abstract

The retina is a complex, multilayered tissue responsible for the perception of visual stimuli from the environment. Contrary to mammals, the capacity for postnatal eye growth in fish and amphibians, and to a lower extent in birds, is coordinated with a progenitor population residing in the ciliary marginal zone (CMZ) at the retinal peripheral margin. However, little is known about embryonic retinogenesis and postnatal retinal growth in squamates (lizards, snakes), despite their exceptional array of ecologies and ocular morphologies. Here, we address this gap by performing the first large‐scale study assessing both ontogenetic and adult changes in the stem/progenitor activity of the squamate peripheral retina. Our study reveals for the first time that squamates exhibit a source of proliferating progenitors persisting post embryogenesis in a newly identified retinociliary junction anteriorly adjacent to the retina. This region is strikingly similar to the vertebrate CMZ by its peripheral location and pseudostratified nature, and shares a common pattern of slow‐cycling cells, spatial differentiation gradient, and response to postnatal ocular growth. Additionally, its proliferative activity varies considerably among squamate species, in correlation with embryonic and postnatal differences in eye size and growth. Together our data indicate that squamates possess a proliferative peripheral retina that acts as a source of progenitors to compensate, at least in part, for postnatal ocular growth. Our findings also highlight the remarkable variation in activity and location of vertebrate retinal progenitors, indicating that the currently accepted scenario of reduced CMZ activity over the course of evolution is too simplistic.

## 1 INTRODUCTION

The eyes of vertebrates, despite possessing a similar overall appearance, structure, and physiology, differ widely in architecture and scaling, which is well related to environmental factors and life characteristics. Within the eye, the neural retina is responsible for perception of visual stimuli from the environment. It develops from the neural ectoderm through early optic cup stages, and forms a complex multilayered tissue lining the posterior inner surface of the eye. Adjacent to the neural retina, another structure derived from the neural ectoderm, called the ciliary body (CB), controls visual accommodation through mechanical lens adjustment and secretes the aqueous humor of the vitreous body (Forrester, Dick, McMenamin, Roberts, & Pearlman, 2016; Tortora & Derrickson, 2012). Vision is a crucial sensory modality in most vertebrates, and although visual performance of the eye also depends on several cellular and biochemical aspects (Hughes, 1977; Walls, 1942), eye size largely contributes to visual acuity and sensitivity (Caves, Sutton, & Johnsen, 2017; Hughes, 1977; Kiltie, 2000; Land & Nilsson, 2012; Walls, 1942). Consequently, various studies assessing the allometry and scaling of vertebrate eye size at different taxonomic levels have correlated adult eye size with various parameters of behavioral ecology such as locomotor behavior, habitat, light conditions, and daily activity patterns (Hughes, 1977; Liu, Ding, Lei, Zhao, & Tang, 2012; Schmitz & Higham, 2018; Werner, 1969; Werner & Seifan, 2006).

A series of complex developmental processes must be carefully orchestrated for the vertebrate eye to form and function correctly. For example, the growth of the retina, lens, and other ocular components must be coordinated to ensure well‐matched dimensions and ultimately optimal visual performance. In vertebrates with lifelong ocular growth such as teleost fish, sharks, and amphibians, a circumferential ring of nonpigmented cells at the extreme periphery of the retina, the so‐called ciliary marginal zone (CMZ), cooperates with central retinal progenitor cells to generate all cell types of the mature retina. The CMZ is maintained throughout adulthood in these “lower” vertebrate groups, thus producing new retinal neurons to accommodate for continuous eye growth. Importantly, however, studies investigating the peripheral retina in amniotes have suggested that both the presence and multipotency of CMZ cells have been diminished through the course of evolution from fish to mammals (Kubota, Hokoc, Moshiri, McGuire, & Reh, 2002). Indeed, a proliferative CMZ was identified at the peripheral retinal margin (RM) in postnatal birds and turtles, but with a more transient (prominent during early postnatal development) and limited neurogenic capacity primarily restricted to amacrine and bipolar cells (Dunlop et al., 2004; Fischer & Reh, 2000; Kubota et al., 2002; Todd et al., 2016). In adult mammals, while a few rare proliferating cells were observed in marsupials such as the opossum in the CB epithelium and RM (Kubota et al., 2002), it is commonly accepted that placental mammals do not have any proliferating RM (Ahmad, Tang, & Pham, 2000; Kubota et al., 2002). As a result, postnatal ocular growth in mammals is believed to be coincident with passive stretching rather than addition of new neurons at either the margin or the central region of the retina (Kuhrt et al., 2012). Nonetheless, a proliferative proximal CMZ has been recently identified during embryogenesis in mice (Bélanger, Robert, & Cayouette, 2017; Marcucci et al., 2016), and dissociated cells from the pigmented epithelium (PE) of CB in several eutherian mammals such as rodents, rabbit, porcine, and human, have been shown to adopt some stem cell‐like properties in culture (Ahmad et al., 2000; Coles et al., 2004; Fernández‐Nogales, Murcia‐Belmonte, Chen, & Herrera, 2019; Tropepe et al., 2000). Furthermore, a proliferative nonpigmented CB region immediately adjacent to the retina has been observed in adult primates and genetically altered mouse lines in vivo (Fischer, Hendrickson, & Reh, 2001; Kiyama et al., 2012; Martínez‐Navarrete, Angulo, Martín‐Nieto, & Cuenca, 2008; Moshiri & Reh, 2004; Reh & Fischer, 2006), thus indicating that the CB might also serve as a putative retinal stem cell zone.

Previous comparative studies are consistent with a gradual decrease of the CMZ or proliferating peripheral retina in vertebrate evolution. However, little is yet known about retinogenesis and morphogenesis of retinal tissues in the squamate group of reptiles (i.e., lizards and snakes), which occupies a key phylogenetic position within amniotes. In particular, whereas the precise anatomy of the reptilian eye has been well described over the past decades, only one recent study directly compared the activity of the peripheral retina in squamates, by using a limited taxon sampling (two snakes and one lizard) at an uncertain adult stage (Todd et al., 2016). Interestingly, the absence of proliferating CMZ or RM progenitors in squamates reported in the latter study is in line with older scattered studies focusing on optic nerve regeneration in several adult lizard species (Beazley, Tennant, Stewart, & Anstee, 1998; Casañas et al., 2011; Dunlop et al., 2004). However, squamates represent the second most diversified group of tetrapods, with more than 10,000 species, and are well known to exhibit substantial variation in eye size and postnatal growth throughout their lifespan (Hallmann & Griebeler, 2018; Shine & Charnovt, 1992). In addition, this geographically widespread group displays an exceptional array of lifestyles, ecologies, and morphological adaptations in eye and visual system related to visual performance and activity pattern (Hall, 2008, 2009; Liu et al., 2012; Werner, 1969; Werner & Seifan, 2006), strongly suggesting that interspecies and/or intraspecies variations in the peripheral retina activity might exist throughout life, as already shown for avians (Fischer & Reh, 2000; Kubota et al., 2002).

Here, we performed the first large‐scale study assessing both ontogenetic and adult changes in the stem/progenitor activity of the squamate peripheral retina, using multiple lizard and snake species from diverse families covering all major groups of squamates. Our findings provide the first evidence of a proliferative peripheral retina that persists into adult squamates, in a pseudostratified region at the retinociliary junction (RCJ) between the retina and the CB. The RCJ progenitors accumulate proliferation markers, express conserved retinal progenitor, and differentiation markers that recapitulate developmental gene expression, and respond to changes in overall body growth rate. Importantly, we further show that the proliferative activity of the RCJ is highly variable among squamate species, in correlation with postnatal variations in ocular size and growth. Altogether, this set of new results coherently and conclusively indicates that squamates possess a proliferative peripheral retina that acts as a source of progenitors to compensate, at least in part, for postnatal ocular growth.

## 2 MATERIALS AND METHODS

### 2.1 Sample collection

All embryonic and postnatal stages of bearded dragons (*Pogona vitticeps*), corn snakes (*Pantherophis guttatus*), and green anoles (*Anolis carolinensis*) were obtained from our animal facility at the University of Helsinki. For embryonic stages, fertilized eggs were incubated on a moistened vermiculite substrate at 29.5°C, and embryos were removed at regular intervals after oviposition to obtain stages covering the whole postovipositional period (about 60 days for *P. vitticeps* and *P. guttatus*, 30 days for *A. carolinensis*). Embryos were staged on the basis of their external morphology according to developmental tables available for lizards and snakes (Boback, Dichter, & Mistry, 2012; Ollonen, Da Silva, Mahlow, & Di‐Poï, 2018). Other adult lizard and snake specimens (21 different species, see Supporting Information) were obtained from private breeders. All reptile captive breedings and experiments were approved by the Laboratory Animal Centre (LAC) of the University of Helsinki and/or the National Animal Experiment Board (ELLA) in Finland (license numbers ESLH‐2007‐07445/ym‐23, ESAVI/7484/04.10.07/2016, and ESAVI/13139/04.10.05/2017).

### 2.2 Computed tomography‐scanning and 3D rendering

High‐resolution computed tomography (CT)‐scans of early embryonic stages of *P. vitticeps*, *A. carolinensis*, and *P. guttatus* at the oviposition stage were obtained from our previous works (Da Silva et al., 2018; Ollonen et al., 2018), while late stages were newly produced at the University of Helsinki or University of Kuopio imaging facilities in Finland using Skyscan 1272 or 1172 microCT, respectively. To visualize eye development, soft tissue of fixed embryos was first stained with 0.6% phosphotungstic acid (PTA) in ethanol, as described before (Metscher, 2009), before scanning using the following parameters: voltage: 59–70 kV; current: 142–167 μA; voxel size: 3.5–10 μm. 3D volume rendering and segmentation of eyes were done manually using Advanced 3D Visualization and Volume Modeling, V5.5.0 (www.fei.com/software/amira-3d-for-life-sciences/, RRID: SCR_007353).

### 2.3 Eye and head length measurements

**Figure 1:**
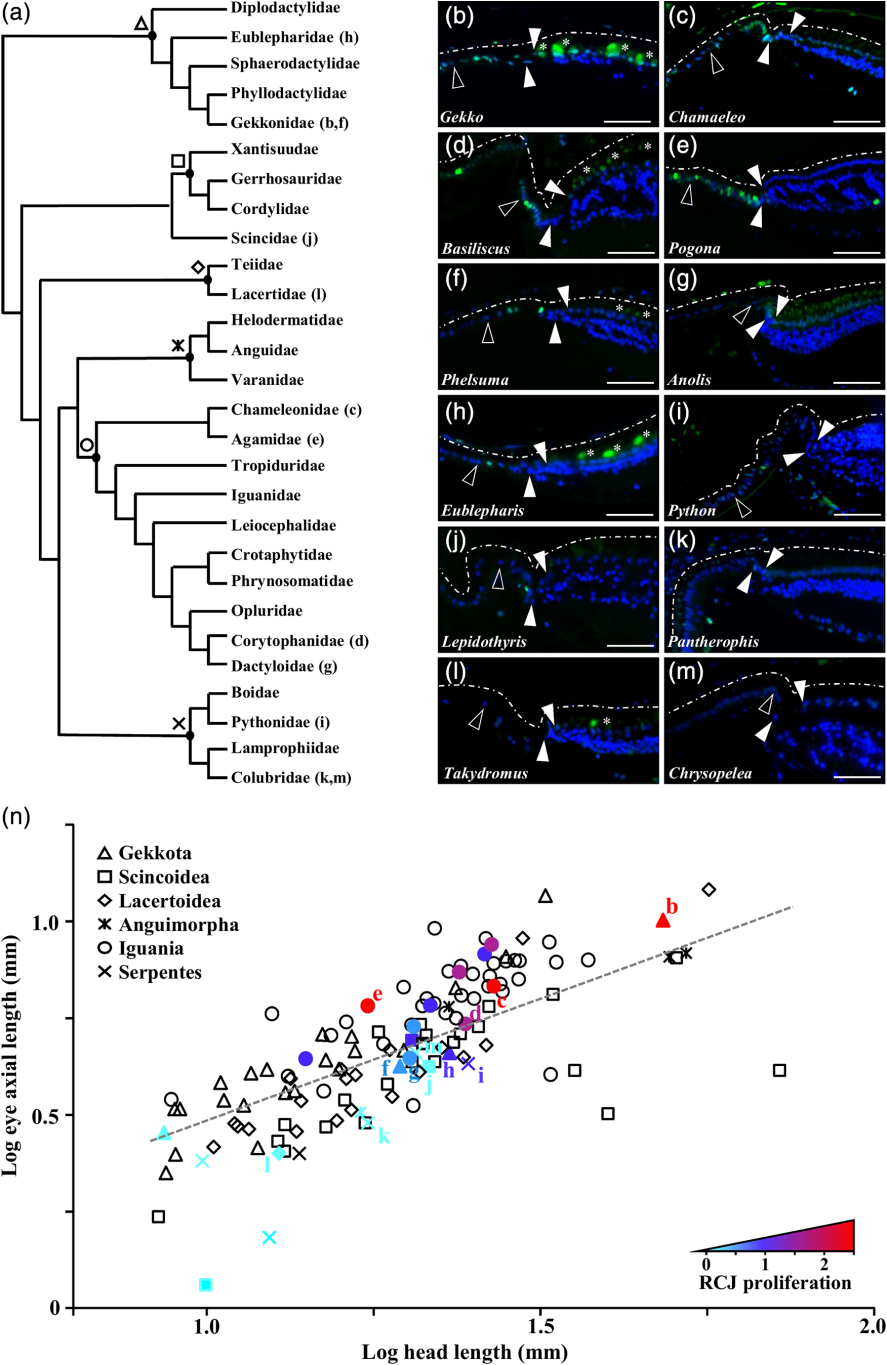
Variability in the proliferation pattern at the peripheral retina in squamates. (a) Simplified phylogeny of squamate families used in this study, adapted from the most inclusive phylogenetic studies available for extant squamate species (Tonini, Beard, Ferreira, Jetz, & Pyron, 2016). Major squamate groups are indicated with different symbols at the nodes: Gekkota (Δ), Scincoidea (□), Lacertoidea (⋄), Anguimorpha (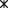), Iguania (○), Serpentes (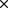). (b–m) Immunohistochemistry with PCNA proliferation marker (green) at the peripheral retina of selected representative juvenile squamates (see indicated position in the phylogenetic tree): Gekko gecko (b), Chamaeleo calyptratus (c), Basiliscus vittatus (d), *Pogona vitticeps* (e), Phelsuma grandis (f), Anolis carolinensis (g), Eublepharis macularius (h), Python regius (i), *Lepidothyris fernandi* (j), *Pantherophis guttatus* (k), *Takydromus sexlineatus* (l), *Chrysopelea ornata* (m). Solid arrowheads delimitate the retinal margin (RM), and open arrowheads indicate the boundary between the monolayered ciliary nonpigmented epithelium (NPE) and the retinociliary junction (RCJ). Dashed white lines outline the pigmented epithelium. White asterisks indicate autofluorescent retinal photoreceptor cells. Cell nuclei are counterstained with DAPI (blue). Scale bars, 100 μm. (n) Scatter plot showing the relationship between log‐transformed eye axial length and log‐transformed head length in 127 lizard and snake species sampled in all major squamate groups (see legend). The dashed gray line represents the regression line estimated by PGLS for all data points. The exact position of selected representative squamate species with different levels of proliferation at the RCJ (b–m) is indicated. Color gradient reflects the average number of PCNA‐positive proliferating cells counted per RCJ region (RCJ proliferation) in tested squamate species, from light blue (no proliferation) to red (high proliferation). Nontested species are colored black

**Figure 2:**
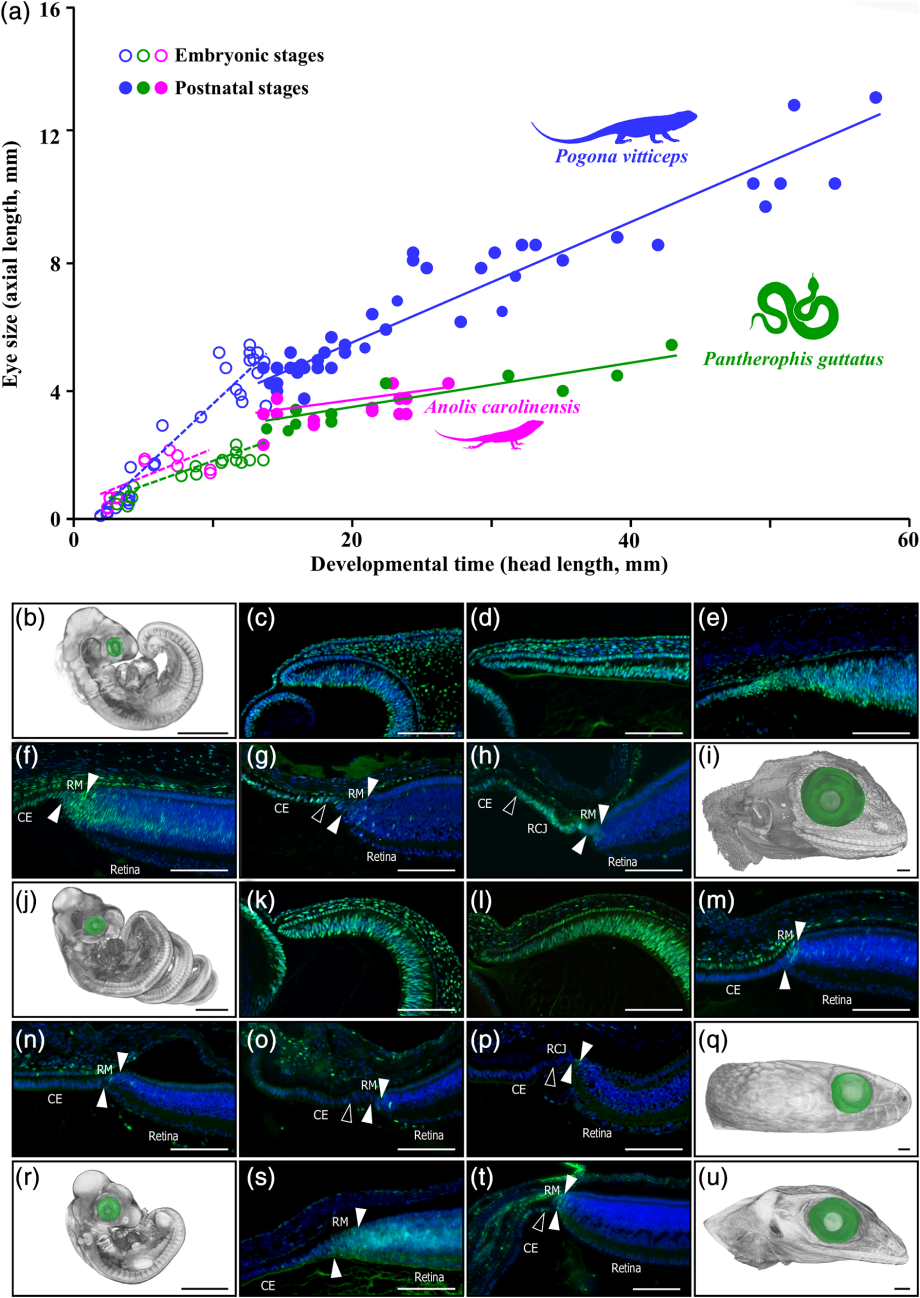
Eye growth and developmental patterns of retinal cell proliferation in selected squamate models. (a) Scatter plot showing the increase in eye axial length (reflecting eye size) over embryonic (open circles) and postnatal (closed circles) development in *P. vitticeps* (blue), *P. guttatus* (green), and A. carolinensis (magenta). Head length rather than age was used as a proxy of developmental time because of considerable variations in growth rate at postnatal stages in a given species (see main text). The postnatal period covers the first two‐thirds of posthatchling lifetime in all species. Colored lines represent species‐specific regression lines for embryonic (dashed lines) or postnatal (solid lines) data points. (b–u) Change in embryonic eye size and retinal cell proliferation in *P*. *vitticeps* (b–i), *P. guttatus* (j–q), and A. carolinensis (r–u), as assessed by 3D‐volume rendering and eye segmentation (green) of PTA‐stained whole‐embryos (b, j, r**)** or embryonic heads (i, q, u) as well as immunohistochemistry with PCNA proliferation marker at the peripheral dorsal retina (c–h, k–p, s, t). Regular developmental stages (every 10 days) covering the entire postoviposition embryonic period, from oviposition (b, c, j, k, r) to hatchling (i, q, u), are shown for each species. Solid arrowheads delimitate the retinal peripheral margin (RM), and open arrowheads indicate the boundary between the monolayered ciliary epithelium (CE) and the retinociliary junction (RCJ) at late embryonic stages. Scale bars, 100 μm (b, i, j, q, r, u) or 1 mm (c–h, k–p, s, t)

Data on head length (HL) and eye axial length (AL) were collected for 127 different squamate species covering all major lineages of squamates (see Supporting Information). Postnatal data in Figure 1n were compiled from both the published literature (Hall, 2008, 2009) and our own measurements on dissected eyes and/or CT‐scans (Supporting Information). Data in Figure 2a covering both embryonic (53 specimens) and postnatal (75 specimens) development of *P. vitticeps*, *A. carolinensis*, and *P. guttatus* were all newly obtained in this study. HL and AL in new specimens were measured anteriorly‐posteriorly from tip of the snout to the posterior end of the external auditory meatus (or posterior end of the braincase when absent in some lizard and snake species) and from the anteriormost portion of the corneal/spectacle surface to the posteriormost portion of the eyeball, respectively, as described previously (Hall, 2008, 2009). Measurements on CT‐scans were performed with Advanced 3D Visualization and Volume Modeling, V5.5.0 (www.fei.com/software/amira-3d-for-life-sciences/, RRID: SCR_007353), by aligning the 3D measurement tool in three orthogonal views within volume renderings of the total head.

### 2.4 Statistical analyses

The relationship between AL and HL in the interspecies data was analyzed with phylogenetic generalized least squares (PGLS) comparative methods in the R package “Caper” v1.0.1 (Orme et al., 2018), using maximum likelihood estimation of parameters for regressions and the most inclusive phylogenetic tree available for extant squamate species (see Figure 1a and Tonini et al., 2016). The phylogenetic signal was estimated as the value *λ* of the residuals, varying between 0 (phylogenetic independence) and 1 (trait evolution under Brownian motion). For intraspecies data, ordinary least squares regressions were performed in XLSTAT software (www.xlstat.com/en/, RRID: SCR_016299). The regression slopes were initially tested for homogeneity, and then regular or phylogenetic analysis of covariance (ANCOVA) was used to assess differences in slopes and/or intercepts between groups, developmental stages, and/or species.

### 2.5 Pulse‐chase labeling experiments

For pulse labeling, 5‐bromo‐2′‐deoxyuridine (BrdU) was administrated twice daily (80 mg/kg body weight) for a period of 7 days by squirting the solution into the mouth. Based on previous vertebrate studies, this duration of pulse labeling is required to detect possible persistent, slow‐cycling retinal progenitors (Kiyama et al., 2012). Animals were euthanized 1, 15, 29, 59, 76, or 113 days after pulse labeling. To minimize differences in body weight increment over the entire course of the experiments, animals were pair‐fed the same amount of diet daily starting 1 week prior to the initial pulse labeling.

### 2.6 In situ hybridization and immunohistochemistry

Eyes were dissected and opened nasally to allow for better penetration during subsequent histological processing. Samples were fixed overnight in 4% paraformaldehyde (PFA) at 4°C before dehydration although a series of alcohol solutions (25, 50, 75, and 100%), paraffin embedding, and sectioning at 7 μm. In situ hybridization (ISH) on paraffin sections was performed as described previously (Gasse, Chiari, Silvent, Davit‐Béal, & Sire, 2015), with minor modifications. Shortly, sections were first rehydrated, and pretreated with proteinase K (Roche) at 37°C for 10 min; then acetylated with 0.25% acetic anhydride in 0.1M triethanolamine buffer for 15 min at RT; and subsequently fixed with 4% PFA for 15 min at room temperature (RT). Finally, sections were hybridized at 60–65°C with digoxigenin (DIG)‐labeled antisense riboprobes corresponding to *P. vitticeps* achaete‐scute family bHLH transcription factor 1 (*Ascl1*, 324 bp), atonal bHLH transcription factor 7 *(Atoh7*, 447 bp), glial fibrillary acidic protein (*Gfap*, 1,030 bp), hes family bHLH transcription factor 1 *(Hes1*, 740 bp), hes family bHLH transcription factor 5 *(Hes5*, 422 bp), neurogenic differentiation 1 (*Neurod1*, 779 bp), neurogenic locus notch homolog protein *1 (Notch1*, 903 bp), orthodenticle homeobox 2 *(Otx2*, 957 bp), paired box 6 (*Pax6*, 654 bp), retinal homeobox gene 1 *(Rx1*, 978 bp), *or* visual system homeobox 2 *(Vsx2*, 1,273 bp). Corresponding sense riboprobes were used as negative controls. After hybridization, sections were washed and incubated overnight at 4°C with anti‐DIG antibodies (1:2,500, sheep polyclonal, Sigma‐Aldrich, cat# 11093274910, RRID: AB_2734716) conjugated with alkaline phosphatase. A staining solution containing 5‐bromo‐4‐chloro‐3‐indolyl phosphate and nitro blue tetrazolium was applied for 1–5 days to visualize hybridization. Finally, slides were mounted using Dako Faramount aqueous medium (Agilent). IHC fluorescent staining was performed as described previously (Di‐Poï & Milinkovitch, 2013), using heat‐induced epitope retrieval (HIER) and overnight incubation at 4°C with primary antibodies known to recognize reptile and/or chicken epitopes: BrdU (1:300, rat monoclonal, Abcam, cat# ab6326, RRID: AB_305426), calretinin (CR; 1:100, rabbit polyclonal, Abcam, cat# ab702, RRID: AB_305702), doublecortin (DCX; 1:200, rabbit polyclonal, Cell Signaling Technology, cat# 4604, RRID: AB_561007), glial fibrillary acidic protein (GFAP; 1:300, mouse monoclonal, LifeSpan, cat# LS‐C88015‐100, RRID: AB_1795608), neuronal‐specific RNA‐binding proteins HuC/D (HuC/D; 1:200, mouse monoclonal, Thermo Fisher Scientific, cat# A‐21271, RRID: AB_221448), proliferating cell nuclear antigen (PCNA; 1:200, mouse monoclonal, BioLegend, cat# 307901, RRID: AB_314691), SRY (sex determining region Y)‐box 2 (SOX2; 1:400, rabbit polyclonal, Abcam, cat# ab97959, RRID: AB_2341193), and SRY‐box 9 (SOX9; 1:400, rabbit polyclonal, Millipore, cat# AB5535, RRID: AB_2239761). Last, incubation with Alexa Fluor‐conjugated secondary antibodies (Alexa Fluor‐488: 1:500, goat anti‐rabbit IgG, Thermo Fisher Scientific, cat# A‐11008, RRID: AB_143165; Alexa Fluor‐568: 1:500, goat anti‐rabbit IgG, Thermo Fisher Scientific, cat# A‐11011, RRID: AB_143157) was performed for 1 hr at RT, and slides were mounted with Fluoroshield mounting medium (Sigma‐Aldrich) containing 4′,6′‐diamidino‐2‐phenylindole (DAPI). For ISH‐IHC double labelings, ISH was performed first followed by IHC omitting the HIER step. Imaging of slides was done using a Nikon Eclipse 90i fluorescence microscope. Fluorescence and bright field images were acquired with Hamamatsu Flash4.0 and Nikon DS‐Fi U3 cameras, respectively, before processing in Adobe Photoshop CC (www.adobe.com/products/photoshop.html, RRID: SCR_014199) using linear levels adjustment.

### 2.7 Quantification of proliferative activity in the RCJ

The RCJ region between the monolayered ciliary epithelium and RM was identified based on morphological and anatomical features, including the pseudostratified columnar epithelia of the RCJ detectable by DAPI nuclear staining, and/or SOX9 immunohistochemistry (IHC) (expression restricted to the RM, see main text). A minimum number of four RCJs per species covering similar longitudinal sectioning planes of the central region of the eye (as assessed by the presence of pupil, lens, optic nerve head, and fovea if possible/present) were selected for quantification. As both the size and nuclei number of the RCJ are highly variable among species in contrast to the overall range of proliferating cell number, proliferative activity was quantified based on the absolute rather than relative number of PCNA‐immunoreactive cell nuclei per RCJ. Central retina was used as a positive control tissue for PCNA IHC in species with nonproliferative RCJ.

## 3 RESULTS

### 3.1 Patterns of cell proliferation in the peripheral retina of adult squamates

The retina of vertebrates such as fish and amphibians grows continuously throughout life via the addition and integration of newly generated neurons at the CMZ. This continuous retinal growth is coordinated with the postnatal growth of the body and contributes to increase ocular size. Squamate reptiles, similar to fish and amphibians, grow throughout their lifespan and thus represent another important model to study postembryonic neurogenesis. Previous studies, however, have so far failed to identify proliferating CMZ or RM progenitors in the few tested adult squamates (Beazley et al., 1998; Casañas et al., 2011; Dunlop et al., 2004; Todd et al., 2016). We first assessed the presence of a proliferating peripheral retina in a larger and more representative panel of lizards and snakes, including 24 different species covering all major groups of squamates (Figure 1a): Gekkota (*n* = 4), Scincoidea (*n* = 3), Lacertoidea (*n* = 1), Anguimorpha (*n* = 1), Iguania (*n* = 10), and Serpentes (*n* = 5). Strikingly, in most specimens tested, immunohistochemical detection of proliferating cell nuclear antigen (PCNA) proliferation marker clearly indicates cell proliferation at the peripheral retina in a region immediately adjacent to the multilayered retina (Figure 1b–m). However, comparative visual inspection of these proliferation patterns reveals marked differences among squamate species in the overall number of proliferating cells, ranging from high (Figure 1b–e) to low (Figure 1f–k) or even absence of detection (Figure 1l,m). In addition, while some proliferating cells are evident in the RM (Figure 1b–e), most of the proliferative activity extends anteriorly into the nonpigmented epithelium (NPE) directly adjacent to the RM (Figure 1b–k). Based on nuclei organization, this proliferating region, which we refer to as the retinociliary junction or RCJ, appears as a pseudostratified columnar epithelium contiguous with, but distinct from the monolayered NPE of the CB. The RCJ region is of variable size, depending on species, and is particularly pronounced in highly proliferative species such as *Chamaeleo calyptratus* and *P. vitticeps* (Figure 1c,e).

In fish and amphibians, and to a lesser extent in avians, the proliferative RM persists into adulthood, thus contributing to the postnatal increase in eye size. To first assess the overall variation in adult eye size across squamates, we compiled published and newly obtained data on both head length (HL) and eye axial length (AL) for 127 lizard and snake species covering all major lineages (Supporting Information). HL was preferred to body (snout‐vent) length in our comparative studies because of the elongated body plan in snakes and some lizard species. As expected, our combined HL and AL data indicate substantial variation in adult eye size both in absolute and relative terms (Figure 1n), and PGLS regression analysis reveals a significant hypoallometric correlation between AL and HL in the whole squamate dataset (*R* ^2^ = 0.66; *p*‐value = 0.0008). Considering the variation in relative eye size among adult lineages, we next explored the potential correlations between relative eye size and proliferative activity at the peripheral retina. As shown in Figure 1n, quantitative comparisons of the average number of PCNA‐positive cells at the RCJ in our sampled species (Supporting Information) confirm our previously observed interspecies variation in proliferative activity. In particular, snakes systematically show a relatively low number of proliferating cells at the RCJ, whereas among lizards substantial variation exists in proliferative activity—from high levels in agamid species such as *P. vitticeps* (see also Figure 1e) to low levels or even absence of detection in species such as *Takydromus sexlineatus* (see also Figure 1l). In addition, all tested species with low (<0.5 positive cells/RCJ) or high (>1.5 positive cells/RCJ) proliferative RCJ exhibit AL lying below or above the regression line, respectively, suggesting a positive correlation between RCJ proliferative activity and relative eye size. This correlation was indeed confirmed using phylogenetic ANCOVA, which indicates that the relative AL in low‐proliferating species (<0.5 positive cells/RCJ) is significantly smaller than in other species (*p*‐value (slope) = 0.32; *p*‐value (intercept) = 0.03). Altogether, this multispecies comparison indicates for the first time that squamates, including both lizards and snakes, exhibit postnatal cell proliferation at the peripheral retina in a RCJ region between the retina and the CB. Furthermore, the major interspecies variations in RCJ proliferative activities correlate with relative eye size, suggesting the contribution of RCJ to postnatal ocular growth in squamates.

### 3.2 Comparative patterns of growth and retinal cell proliferation in the developing eye

In most squamate taxa, like in fish, turtles, and most amphibians, growth continues throughout life at a steadily decreasing rate (Congdon, Gibbons, Brooks, Rollinson, & Tsaliagos, 2013; Dutta, 1994; Hallmann & Griebeler, 2018; Shine & Charnovt, 1992). In this context, the overall size and growth rate of the eye and its components, including the retina, change throughout ontogeny to achieve optimal visual perception. To assess ontogenetic changes in squamate eye development, we compared embryonic and postnatal eye growth in three model species showing different RCJ proliferative activities at adult stage (see Figure 1n), including one high‐proliferating lizard (*P. vitticeps*; Figure 1e), one low‐proliferating lizard (*A. carolinensis*; Figure 1g), and one low‐proliferating snake (*P. guttatus*; Figure 1k). Head length rather than age was used as a proxy of developmental time in our analyses because of considerable, nonage related intraspecies variations in growth rate at postnatal stages (see Figure 4a); such variations in reptilian growth rate have already been shown to be associated with differences in food availability, social factors, and thermoregulation time (Andrews, 1982). At the earliest embryonic time point investigated (oviposition time, referred to as 0 days postoviposition [dpo]), morphogenesis of the neural retina has already started and both the optic cup and lens vesicle have already formed in *P. vitticeps* (Figure 2b,c), *P. guttatus* (Figure 2j,k), and *A. carolinensis* (Figure 2r). Interestingly, however, *P. vitticeps* exhibit a significantly higher relative embryonic growth rate of the eye compared to other species (*p*‐values (slopes) < 0.028), as revealed by pairwise comparisons of species‐specific regressions using ANCOVA analysis (Figure 2a). This increased embryonic growth leads to a more than 30‐fold increase in AL and ultimately to larger absolute eye size in *P. vitticeps* at hatching (Figure 2a,i). Postnatally, the higher rate of relative eye growth is maintained in *P. vitticeps* (ANCOVA: *p*‐values (slopes) < 0.006), while *P. guttatus* and *A. carolinensis* show relatively similar eye size relative to HL throughout embryonic and postnatal periods (ANCOVA: *p*‐values (slopes) > 0.84; *p*‐values (intercepts) > 0.47).

To investigate the potential role of the RCJ in lifelong ocular/retinal growth in our selected models, we next inspected the proliferative activity of the retina during postovipositional embryonic development by IHC with PCNA. As shown for other vertebrate species, a widespread proliferation throughout the optic cup is visible at early oviposition stages in squamates (0 dpo; Figure 2c,k). At later embryonic stages, a gradient of neurogenesis appears, as proliferation ceases in the center of the optic cup and becomes more confined toward the peripheral retina. While this general pattern applies to *P. vitticeps* (Figure 2d–h), *P. guttatus* (Figure 2l–p), and *A. carolinensis* (Figure 2s,t), both the amount and timing of proliferation differs between species. In *P. vitticeps*, the first lamination can already be noticed in the central retina at 20 dpo (data not shown), while the retinal periphery remains nonlaminated and highly proliferative at early embryonic stages (Figure 2c–e). Over this period, no clear boundary between cells of the presumptive developing CB and neural retina is apparent. At mid‐development (30 dpo), retinal lamination has proceeded to the periphery, as evident from the plexiform layers now delimitating the posterior part of the RM, and both the RM and a thin neuroblastic layer in the presumptive inner nuclear layer (INL) are still highly proliferative (Figure 2f). This remaining proliferative peripheral area will progressively diminish over late embryonic stages and becomes confined to the RM, the presumptive RCJ directly adjacent to the RM, and the CB NPE (Figure 2f–h). Shortly before hatching, only a well‐distinguishable pseudostratified epithelium at the RCJ between the low‐proliferative RM and monolayered CB NPE remains highly proliferative (Figure 2h). Interestingly, similar progressive lamination and proliferative restriction at the peripheral retina also happen during embryogenesis in *P. guttatus* and *A. carolinensis*. However, these processes appear much faster than in *P. vitticeps*, and already at mid‐embryonic development the peripheral retina displays noticeably fewer proliferating cells (Figure 2m–o,s,t). Similarly, in contrast to *P. vitticeps*, the RCJ near hatchling time is reduced in size and nonproliferative or low‐proliferative (Figure 2p). Altogether, our results indicate a progressive restriction of proliferation toward the RM and then RCJ region over the course of embryogenesis, suggesting that the proliferative RCJ observed in adult squamates already emerges at late embryonic stages. In addition, the expanded proliferation pattern observed in the developing peripheral retina of *P. vitticeps* is coherent with the increased growth rate and relative eye size of this species already starting from embryonic stages.

### 3.3 Molecular characterization of the embryonic peripheral retina

**Figure 3:**
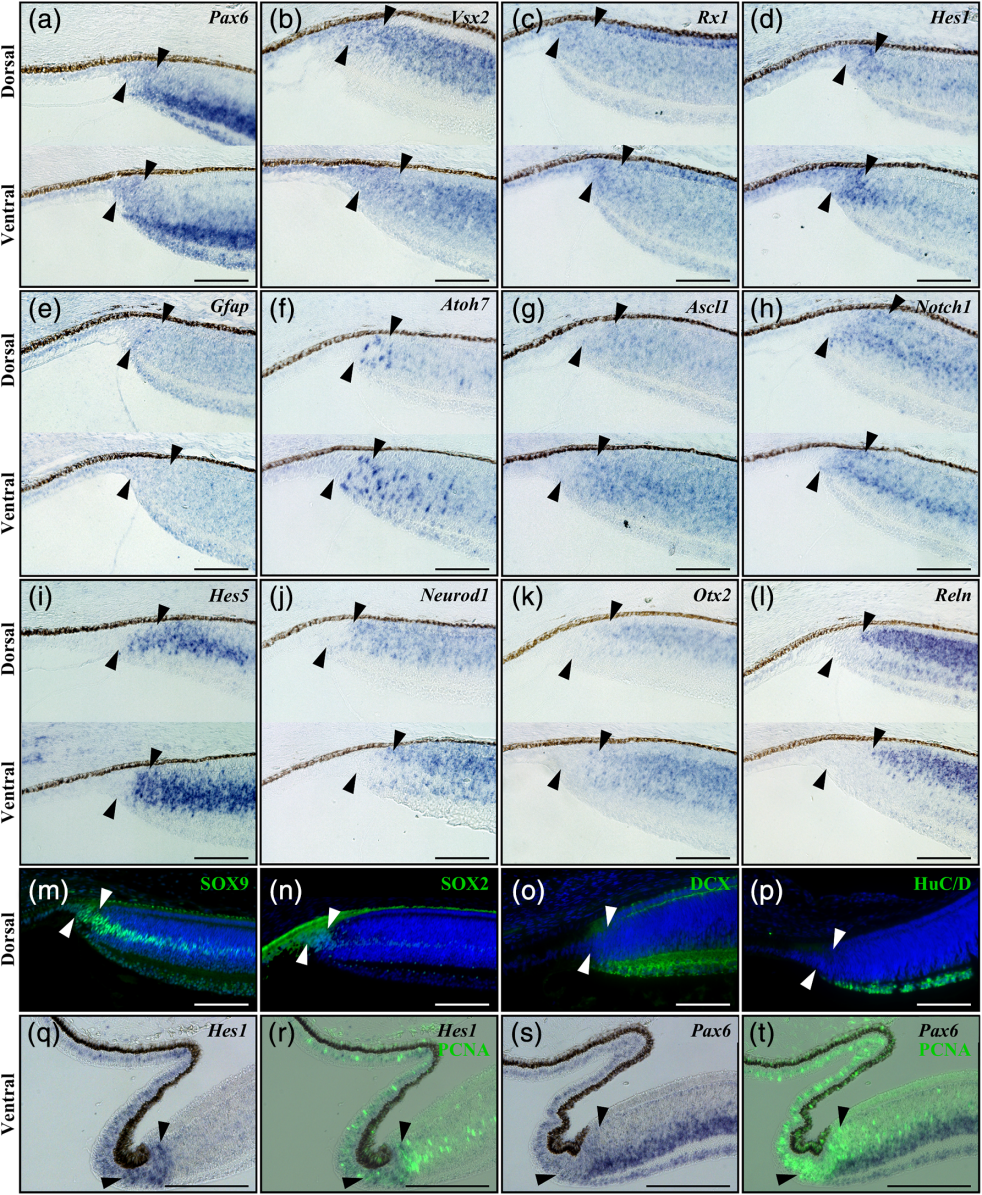
Expression pattern of conserved retinal markers in the peripheral retina of *P. vitticeps* embryos. (a–l) In situ hybridization showing the expression of various markers of retinal development at both dorsal (dorsal) and ventral (ventral) regions of the peripheral retina in *P. vitticeps* embryos at 30 dpo: *Pax6* (a), *Vsx2* (b), *Rx1* (c), *Hes1* (d), *Gfap* (e), *Atoh7* (f), *Ascl1* (g), *Notch1* (h), *Hes5* (i), *Neurod1* (j), *Otx2* (k), and *Reln* (l). (m–p) Immunohistochemistry with SOX9 (m), SOX2 (n), DCX (o), and HuC/D (p) retinal markers (green) at the peripheral dorsal retina in *P. vitticeps* embryos at 30 dpo. Cell nuclei are counterstained with DAPI (blue). (q–t) In situ hybridization showing either the single expression of *Hes1* (q) and *Pax6* (s) retinal markers (blue) or their codetection with PCNA proliferation marker (green) by immunohistochemistry (r, t) at the peripheral ventral retina in *P. vitticeps* embryos at 40 dpo. Solid arrowheads delimitate the retinal peripheral margin. Scale bars, 100 μm

To further investigate embryonic retinal growth in squamates, and more particularly the formation and patterning of the proliferative RM and RCJ, we characterized at the molecular level the peripheral retina in *P. vitticeps* at mid‐embryonic development (30 dpo). At this stage, retinal differentiation has proceeded to the periphery and the proliferative RM, presumptive RCJ, and laminated retina are all apparent, thus allowing investigation of expression patterns with defined spatial resolution. Indeed, as already shown for the vertebrate CMZ, this spatial expression gradient is presumed to reflect the temporal expression sequences of stem/progenitor and differentiation markers with respect to retinal development. We thus examined, with ISH and/or IHC, the expression patterns of classical conserved markers of retinal development, including stem/progenitor, proneural, and postmitotic neuronal differentiation markers. Similar to retinal stem/progenitor cells in the developing retina of other vertebrates (Fischer & Reh, 2000; Perron, Kanekar, Vetter, & Harris, 1998; Raymond, Barthel, Bernardos, & Perkowski, 2006; Todd et al., 2016), stem/progenitor markers such as *Pax6*, *Vsx2*, *Rx1*, and *Hes1* (mRNA levels, Figure 3a–d) as well as SOX9 and SOX2 (protein levels, Figure 3m,n) are all coexpressed at low levels in the RM, while their expression becomes relatively higher and exclusive to particular layers in the differentiated retina (Figure 3a–d,m,n). A similar expression pattern was observed for the glial marker *Gfap*, which is restricted to a few cells along the retinal edge of the RM (Figure 3e) and to presumptive Müller glia cells in the central retina (data not shown). Importantly, the observed specificity of the different tested markers to particular mature retinal cell types and layers outside the RM is relatively well conserved with previous reports in vertebrates, including reptiles (Romero‐Alemán, Monzón‐Mayor, Santos, Lang, & Yanes, 2012; Simoniello et al., 2014; Todd et al., 2016). In addition, despite a slight developmental delay at the peripheral ventral retina, when compared to its dorsal counterpart, similar spatial patterns of gene expression were observed in both regions (Figure 3a–e). Remarkably, with the exception of SOX9 that appears more restricted to the RM (Figure 3m), expression of all tested stem/progenitor markers extends anteriorly to the RM toward the presumptive RCJ and NPE. In the latter regions, our double ISH‐IHC stainings confirm that the majority of proliferating PCNA‐positive cells coexpress stem/progenitor markers such as *Hes1* and *Pax6* (Figure 3q–t), thus indicating the presence of an expanded proliferative zone with retinal progenitors. In the presumptive retinal INL, in contrast, progenitor markers such as *Hes1* are not detected in the layer of proliferating neuroblasts. Consistent with a spatial gradient of differentiation at the peripheral retina, proneural genes known to endow progenitors with a neuronal fate, including *Atoh7* and *Ascl1*, are mainly detected in the posteriormost part of the proliferative RM (Figure 3f,g), and both genes then stretch into the peripheral retina INL while tapering off toward the central retina. A similar expression pattern is observed for *Notch1*, a well‐known inhibitor of proneural genes, and its downstream target *Hes5* (Figure 3h,i). Finally, key signaling factors involved in neuronal differentiation and/or migration, including *Neurod1*, *Otx2*, and *Reln* (mRNA levels, Figure 3j–l) as well as DCX and HuC/D (protein levels, Figure 3o,p), are barely detected in the proliferating RM, except for a few cells expressing early neuronal differentiation markers such as *Neurod1* at the posteriormost part of the RM, and their expression rather stretches throughout the postmitotic laminated retina (Figure 3j–l). Altogether, these expression profiles highlight an anteroposterior gradient in the spatial ordering of genes at the peripheral retina, with stem/progenitor markers being confined to the presumptive RCJ and RM and early differentiating markers more posterior within or directly adjacent to the RM at the onset of retinal lamination, confirming that the RCJ is gradually formed during embryonic development.

### 3.4 Growth‐dependent activity of the postnatal peripheral retina in *P. vitticeps*

**Figure 4:**
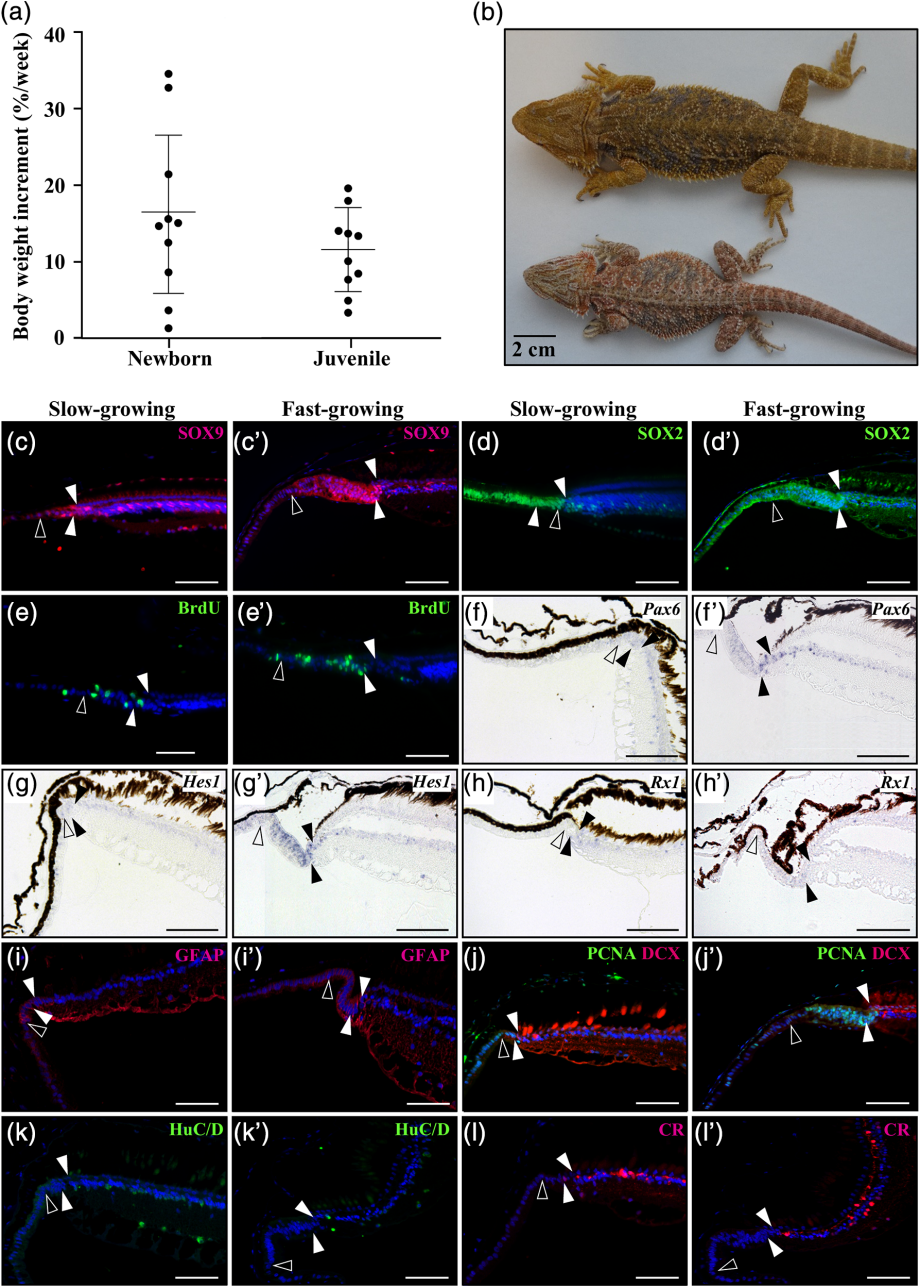
Effect of body growth rate on peripheral retina activity in *P. vitticeps* lizards. (a) Variability in the percentage of total body weight increment per week in newborn (0–3 months after hatchling) and juvenile (3–12 months) *P. vitticeps*. Data are shown as mean ± SEM, *n* = 10 per group. (b) Dorsal views of representative slow‐growing (bottom, body weight of about 80 g) and fast‐growing (top, about 180 g) *P. vitticeps* males at 2‐years old. (c–l’) Immunohistochemistry (c–e’, i, i’, k–l’), double immunohistochemistry (j, j’), or in situ hybridization (f–h’) showing the expression of various retinal progenitor (SOX9, SOX2, *Pax6*, *Hes1*, *Rx1*), proliferation (BrdU, PCNA), and/or differentiation (DCX, GFAP, HuC/D, CR) markers at the peripheral dorsal retina in slow‐growing (c, d, e, f, g, h, i, j, k, l) and fast‐growing (c’, d’, e’, f’, g’, h’, i’, j’, k’, l’) juvenile *P. vitticeps*. The names of retinal markers (color‐coded according to the immunofluorescence signal for protein and BrdU detection) are shown on the top right corner in each panel. Solid arrowheads delimitate the retinal peripheral margin, and open arrowheads indicate the boundary between the monolayered ciliary epithelium and the RCJ. Scale bars, 100 mm

To determine if the identified proliferative RCJ could serve as a source of retinal progenitors beyond embryonic stages, we next characterized the activity and gene expression profiles of the postnatal peripheral retina in *P. vitticeps*. Based on the species‐dependent proliferation patterns observed at the RCJ during both embryonic and adult stages, we further hypothesized that this progenitor activity could be influenced by the overall eye growth that accompany body size increase at postnatal stages (Figures 1n and 2a). To test this hypothesis, we took advantage of the individual variations in body growth rate observed in *P. vitticeps*, particularly in newborn and juvenile animals (0–12 months after hatchling, Figure 4a), which lead to considerable differences in body weight and absolute eye size at similar adult age (Figure 4b). Comparisons of the peripheral retina in slow‐growing and fast‐growing juveniles, based on a cut‐off of 10% body weight increment per week (Figure 4a), immediately reveal a large anteroposterior expansion as well as an increase in the apical‐basal thickness of the pseudostratified RCJ in fast‐growing animals (see, e.g., Figure 4c,c’). Coherent with this finding, the absolute number of cells expressing stem/progenitor markers is also markedly increased in this RCJ region (Figure 4c–d’,f–i’). Likewise, BrdU‐positive proliferating cells after 1 week of pulse labeling are more abundant in the RCJ of fast‐ versus slow‐growing animals (Figure 4e,e’). However, the spatial gradient of gene expression observed during embryonic retinal development is rather conserved between the two groups. Regardless of individual growth rate, all progenitor markers but SOX2 are widely colocalized in the RCJ and RM, while being virtually absent from the monolayered CB NPE (Figure 4c–i’). These expression patterns are coherent with our IHC with BrdU and PCNA proliferation markers, which also indicates that proliferating cells are abundant in the RCJ and more scattered along the entire length of the CB NPE (Figure 4e,e’,j,j’). In addition, late postmitotic neuronal markers of amacrine and ganglion cells such as HuC/D and CR are only detected in the differentiated laminated retina (Figure 4k–l’), whereas early neuronal differentiation markers such as DCX are expressed in a few proliferating cells at the posteriormost part of the RM (Figure 4j,j’), thus indicating the generation of new neurons.

**Figure 5:**
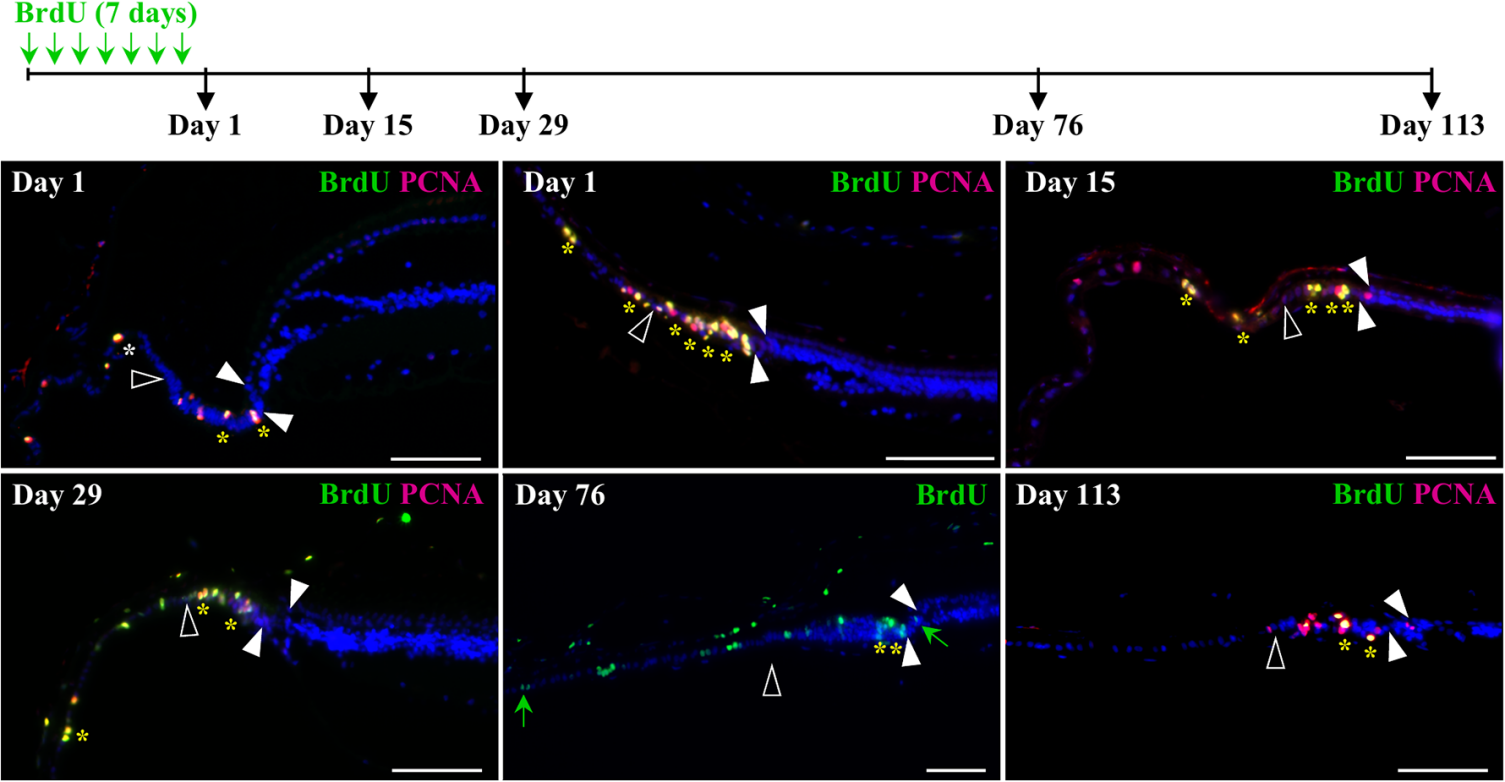
Identification and localization of putative slow‐cycling stem cells in the peripheral retina of *P. vitticeps*. The top schematic drawing depicts the experimental strategy and BrdU pulse‐chase time points (black arrows). Retinal tissues were collected in adult (>2‐years old, top left panel) and/or juvenile (<1‐year old, other panels) *P. vitticeps* at 1, 15, 29, 76, or 113 days after the first week of BrdU feeding, and sections from the peripheral dorsal retina were processed for double immunohistochemistry against BrdU (green) and PCNA (red). Solid arrowheads delimitate the retinal peripheral margin, and open arrowheads indicate the boundary between the monolayered ciliary epithelium and the RCJ. The localization and number of yellow asterisks reflect the position and relative abundance of BrdU/PCNA double‐positive cells, respectively, in the ciliary epithelium, RCJ, and retinal margin. Only BrdU labeling is shown after 76 days of chase to better highlight the diluted BrdU‐labeled cells scattered outside the RCJ within both the ciliary body and the retina (green arrows). Cell nuclei are counterstained with DAPI (blue). Scale bars, 100 μm

To further assess the localization and behavior of putative slow‐cycling, label‐retaining stem/progenitor cells in the peripheral retina of *P. vitticeps*, we next performed BrdU pulse‐chase assays at different postnatal stages (Figure 5). As predicted, immediately following the BrdU‐pulse (Day 1), proliferative cells colabeled with BrdU and PCNA were found throughout the pseudostratified RCJ in juvenile animals, but also in all tested adult stages covering the first two‐thirds of postnatal lifetime (Figure 5 and data not shown), suggesting the maintenance of a proliferative RCJ throughout the lifespan. During the chase period, while BrdU/PCNA‐double positive cells still remain in the RCJ up to Day 113, more diluted BrdU‐labeled cells are found away from the RCJ at the posterior edge of the RM and in the CB NPE (Days 29 and 76), indicating the presence of both cell division and bidirectional migration into the CB and retina after BrdU uptake. Positive cells become eventually undetectable in the laminated retina and CB, likely as a result of BrdU dilution through cell divisions (Day 113), suggesting that progenitors at the RM are differentiating and integrating rather slowly into the retina, as already observed in other vertebrates (Fischer & Reh, 2000; Kiyama et al., 2012; Kubota et al., 2002; Marcus, Delaney, & Easter, 1999). Finally, the persistence of BrdU/PCNA‐double positive cells in the RCJ after more than 16 weeks of chase (Day 113) clearly supports the presence of slow‐cycling cells in the most peripheral zone of the peripheral retina, as similarly reported in the distal domain of the CMZ in other vertebrates (Centanin, Hoeckendorf, & Wittbrodt, 2011; Raymond, Barthel, Bernardos, & Perkowski, 2006; Wan et al., 2016; Xue & Harris, 2012).

Altogether, these results demonstrate the existence of slow‐cycling stem/progenitor cells at the RCJ as well as the maintenance of a spatial gradient of neuronal differentiation, largely recapitulating the dynamic gene expression pattern observed during embryonic development, in the postnatal peripheral retina of *P. vitticeps*. In addition, our data strongly suggest that this stem/progenitor zone constitutes a source for continued neurogenesis contributing to postnatal retinal growth.

### 3.5 Comparative characterization of the peripheral retina in *P. guttatus*

**Figure 6:**
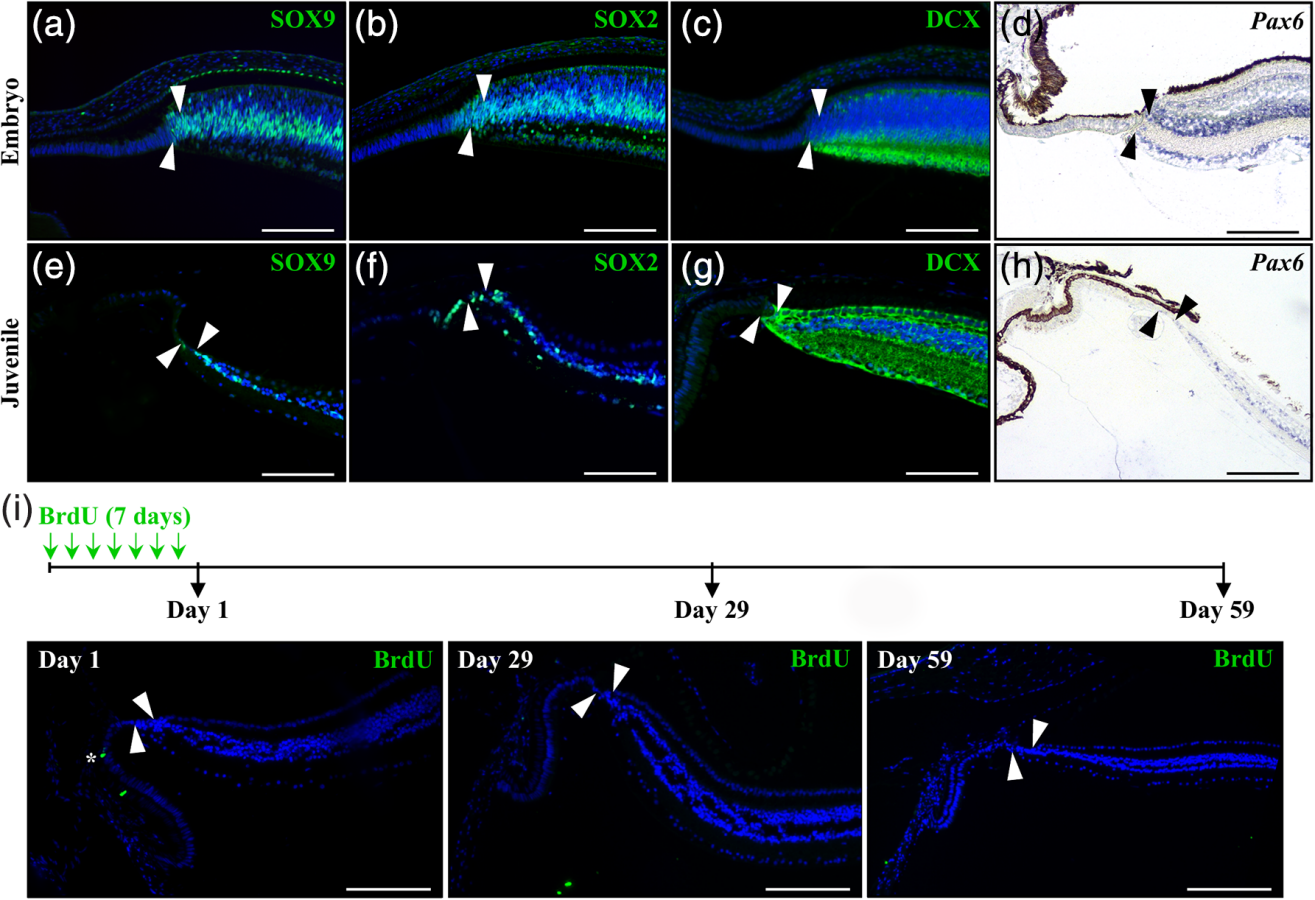
Expression pattern of retinal and proliferation markers in the peripheral retina of *P. guttatus*. (a–h) Immunohistochemistry (a–c, e–g) or in situ hybridization (d, h) showing the expression of various retinal stem cell (SOX2, SOX9, *Pax6*) or differentiation (DCX) markers at the peripheral dorsal retina in *P. guttatus* at both embryonic (12 dpo, a–c; 20 dpo, d) and juvenile (<1‐year old; e–h) stages. The names of retinal markers (color‐coded according to the immunofluorescence signal for protein detection) are shown on the top right corner in each panel. Only the retinal peripheral margin is delimited (solid arrowheads), as the RCJ is strongly reduced in snakes. (i) The top schematic drawing depicts the experimental strategy and BrdU pulse‐chase time points (black arrows). Retinal tissues were collected in newborn (<3‐months old) *P. guttatus* chased for 1, 29, or 59 days from the first week of BrdU feeding, and sections from the peripheral dorsal retina were processed for immunohistochemistry with BrdU (green). Solid arrowheads delimitate the retinal peripheral margin, and the white asterisk indicates one BrdU‐positive cell in the ciliary epithelium (Day 1, left panel). Cell nuclei are counterstained with DAPI (blue). Scale bars, 100 μm

In contrast to *P. vitticeps*, the RCJ in juvenile and adult snakes is strongly reduced in both size and proliferative activity (see Figure 1). Coherent with this, our molecular characterization of the RCJ in *P. guttatus* indicates major differences in the expression patterns of stem/progenitor and differentiation markers at both embryonic (Figure 6a–d) and postnatal (Figure 6e–h) stages. In particular, whereas SOX2 expression expands beyond the RM in a narrow pseudostratified region reminiscent of a RCJ anteriorly adjacent to the RM (Figure 6b,f), SOX9 is not detected in the latter region but rather strictly delimitates the anterior border of the RM (Figure 6a,e). Similar expression patterns were observed for early progenitor (*Pax6*, Figure 6d,h) and differentiation (DCX, Figure 6c,g) markers, which are highly expressed in the laminated retina but not detected at the RCJ and/or RM. Therefore, the RCJ of mid‐embryonic and juvenile *P. guttatus* is characterized by the virtual absence of progenitor markers such as SOX9 and *Pax6*. To further assess the proliferative activity of the peripheral retina in snakes, a BrdU pulse‐chase experiment similar to that of *P. vitticeps* was performed in juvenile *P. guttatus* (Figure 6i). Coherent with the low PCNA immunodetection in the RCJ or RM of this particular species (Figure 1k), BrdU‐positive cells are only detected in both the PE and NPE of CB as well as in extraocular structures such as the spectacle (data not shown) immediately after the BrdU‐pulse (Figure 6i, Day 1). Similarly, no labeled cells were observed in the RCJ or RM later during the chase period (Figure 6i, Days 29 and 59). These results confirm the low RCJ proliferation in *P. guttatus* at both embryonic and postnatal stages, and are in line with the limited postnatal growth and overall expression pattern of peripheral retina markers in this species.

## 4 DISCUSSION

**Figure 7:**
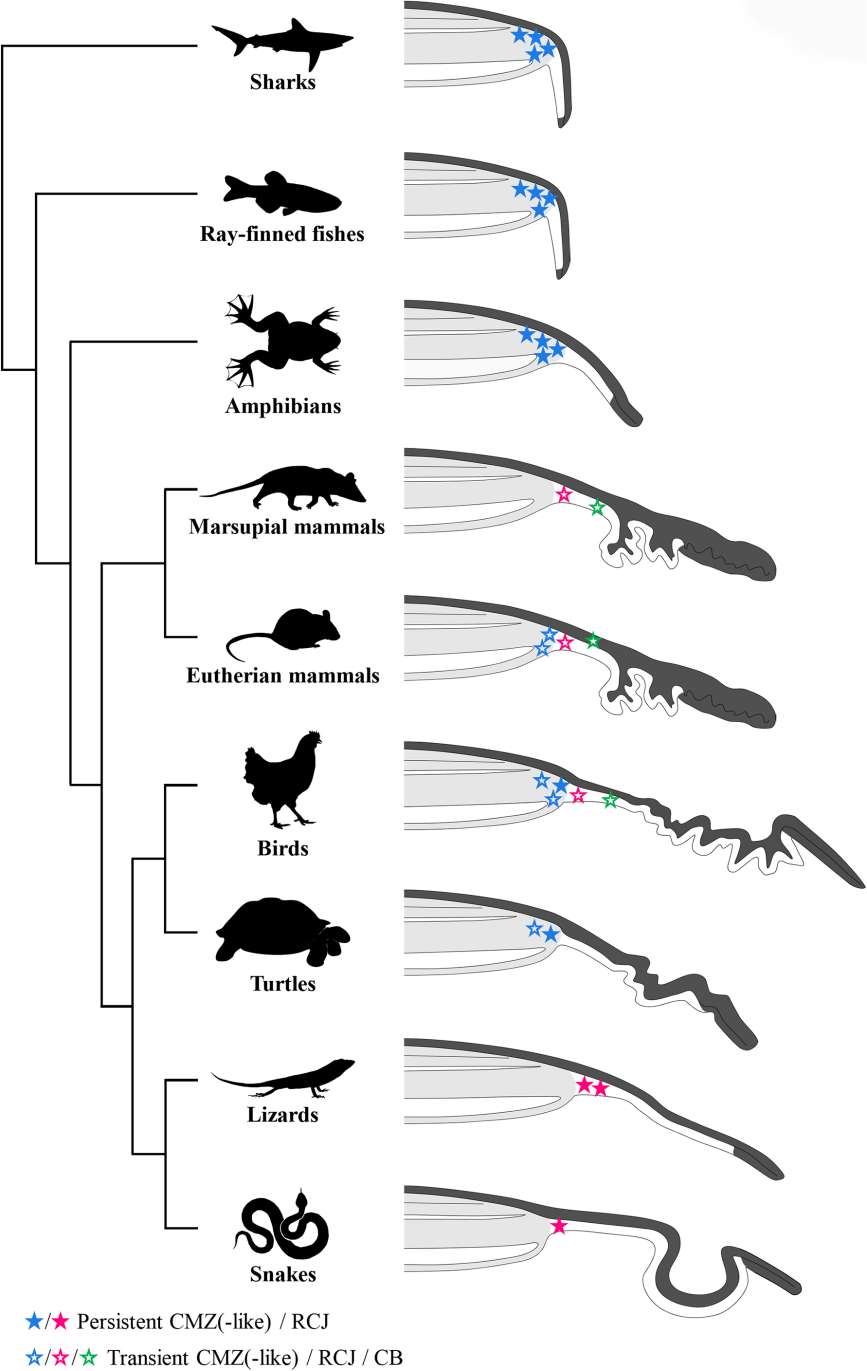
Comparative localization of postnatal proliferation and putative retinal stem cells in the peripheral retina of different vertebrate classes. Phylogenetic relationships of vertebrate lineages with schematic representations of their peripheral retina showing the neural retina (light gray shading), pigmented epithelium (dark gray shading), ciliary body, and iris for some species. The variable localization of postnatal proliferating cells is reflected by both the position and color of stars: ciliary marginal zone (CMZ or CMZ‐like, blue), retinociliary junction (RCJ, magenta), and ciliary body epithelium (CB, green). The postnatal maintenance of proliferating cells at the peripheral retina is shown by solid (persistent) or open (transient) stars, while the relative abundance of postnatal proliferating cells is reflected by the number of stars. The well‐established persistence of proliferating cells (and putative retinal stem cells) in the most peripheral zone of the CMZ in adult sharks, ray‐finned fishes, and amphibians is shown with solid blue stars. A proliferating CMZ‐like region has been identified in postnatal turtles and postnatal birds, although the proliferation pattern is age‐ and/or species‐dependent. In eutherian mammals (including rodents, rabbit, porcine, human), dissociated cells from the pigmented ciliary epithelium have been shown to generate neurospheres, and a proliferative proximal CMZ that disappears at postnatal stages has been recently identified during embryogenesis in mice. The nonpigmented ciliary epithelium of the chick CB also contains quiescent retinal stem cells that proliferate when stimulated with growth factors. Similarly to the situation in lizards and snakes, a proliferative pseudostratified region immediately adjacent to the retina has already been observed in several vertebrate classes, including marsupial mammals, eutherian mammals, and birds. Proliferation at the RCJ is highly variable among lizard and snake species, as indicated by the different number of stars

Following its discovery in amphibians more than 50 years ago (Hollyfield, 1968; Straznicky & Gaze, 1971), the presence of a CMZ capable of mediating postnatal retinal growth has been under investigation in a wide range of vertebrate species under various physiological and pathological conditions. The general consensus arising from these studies is a progressive reduction of the CMZ over the course of vertebrate evolution from fish to mammals (Kubota et al., 2002). However, one of the most specious groups of terrestrial vertebrates—squamate reptiles—has remained largely unexplored except for a recent comparative study investigating a few individuals from three different species (Todd et al., 2016). Here, we demonstrate for the first time that both lizards and snakes contain a source of postnatal proliferating progenitors in a newly identified pseudostratified RCJ region between the retina and the CB. Similarly to the CMZ situation in other vertebrates (Centanin et al., 2011; Raymond, Barthel, Bernardos, & Perkowski, 2006; Wan et al., 2016; Xue & Harris, 2012), our data indicate that the squamate peripheral retina contains slow‐cycling cells at its extreme periphery, and accumulates proliferating progenitors expressing conserved retinal progenitor/stem and differentiation markers in a spatial order that recapitulates developmental gene expression. On the one hand, the peripheral location of squamate progenitors at the RCJ is in agreement with the overall decrease or absence of proliferating cells at the amniote RM (Figure 7); on the other hand, we show here that proliferating progenitors are maintained throughout adulthood in squamates, in contrast to the transient proliferation patterns observed in postnatal birds, mammals, and most likely turtles (Bélanger et al., 2017; Dunlop et al., 2004; Kubota et al., 2002; Marcucci et al., 2016; Reh & Fischer, 2006; Todd et al., 2016). Although the persistence of a proliferative CMZ in turtles is still unclear, it is expected to be age‐ and/or species‐dependent, as proliferating progenitors were not observed in all examined species (Dunlop et al., 2004; Todd et al., 2016). Importantly, our large‐scale comparison further indicates that the postnatal proliferative activity of the RCJ is highly variable among squamates, likely explaining the lack of observations in the eyes of previously published lizard and snake species (Casañas et al., 2011; Dunlop et al., 2004; Todd et al., 2016). Indeed, our analyses confirm the overall reduction of RCJ activity in snakes and in lizard genera such as *Anolis* and *Gallotia*. This variation in adult progenitor activity at the peripheral retina has already been noticed in other vertebrate classes such as avians, with quail having a reduced CMZ compared to chicken, thus highlighting the importance of sampling and species selection in comparative retinal studies.

Similarly to the situation in lizards and snakes, other populations of proliferating stem/progenitor cells anteriorly adjacent to the retina have already been observed at the peripheral retina in several amniote species (Figure 7). In particular, in vivo evidence exists in chicken (Fischer & Reh, 2000), opossum (Kubota et al., 2002), and eutherian mammals such as primates (Martínez‐Navarrete et al., 2008; Tkatchenko, Walsh, Tkatchenko, Gustincich, & Raviola, 2006) and genetically altered mouse lines (Kiyama et al., 2012; Moshiri & Reh, 2004) for a proliferative pseudostratified NPE reminiscent of the squamate RCJ (usually referred to as planoretinal junction or pars plana component of the CB). Although this region in avian and mammalian species is only transient and not clearly defined yet at the molecular level, including its neuroregenerative capacity, these observations still suggest evolutionary conservation of an epithelial stem/progenitor cell population next to the neural retina in amniotes (Figure 7). Consistent with this hypothesis, the adult ciliary epithelium in avians and mammals, when stimulated with growth factors in vivo (Abdouh & Bernier, 2006; Fischer & Reh, 2003), has been shown to contain quiescent retinal stem/progenitor cells and to adopt a pseudostratified configuration resembling the squamate RCJ. Similarly, early embryonic retinal primordia of amphibians and mammals (Fernández‐Nogales et al., 2019; Hollyfield, 1968; Straznicky & Gaze, 1971), including the embryonic proximal CMZ recently identified in mice (Bélanger et al., 2017; Marcucci et al., 2016), show similarities with the squamate RCJ at least in terms of proliferation pattern and pseudostratification, suggesting that they might be equivalent structures only maintained in squamates and in some mammals like primates (see above) at postnatal stages.

Previous vertebrate studies have reported that the proliferative activity of the CMZ, RM, and/or CB contributes to postnatal retinal growth and correlates with increase in ocular size, thus ensuring optimal visual perception. For example, both birds and mammals show determinate growth and reach their final eye size relatively early in their lifetime, coherent with a transient proliferative activity limited to a few weeks after birth at the peripheral margin (Ahmad et al., 2000; Fischer & Reh, 2000; Kubota et al., 2002). In contrast, the retina of fish and amphibians grows continuously throughout life by continuous addition of new cells at the peripheral margin, roughly matching the overall growth of the animal. Similarly to fish and amphibians, and in accord with the continuous growth of most squamates (Hallmann & Griebeler, 2018), our data indicate the persistence of progenitor cells at the RCJ throughout lifetime at least in the tested squamate species. Moreover, the highly variable proliferation patterns observed at the postnatal RCJ within or among lizard and snake species significantly correlate with ocular size and growth rates. Altogether, this set of results coherently indicates that squamates possess a proliferative peripheral retina that acts as a source of proliferating progenitors contributing, at least in part, to postnatal ocular growth. Furthermore, as shown for mammals, birds, and fish, the relatively slow differentiation and integration of progenitors into the squamate retina suggest that alternative mechanisms such as passive retinal stretching might also be involved in compensating for eye growth, a mechanism that might itself be growth‐ and/or species‐dependent. Besides postnatal ocular growth, the maintenance of retinal progenitor cells in squamates might also have potential implications for retinal regeneration and repair. Indeed, persistent CMZ cells in fish and amphibians have been shown to participate to the regeneration process after retinal injury (Reh & Nagy, 1987; Stenkamp, Powers, Carney, & Cameron, 2001), whereas mature Müller glia, the major type of support cell in the retina, have been identified as the cellular source of retinal regeneration in birds and rodents (Fischer & Reh, 2000; Fischer et al., 2001; Karl et al., 2008; Ooto et al., 2004). Future studies investigating the progenitor potential of RCJ and Müller glia in the squamate retina after injury remains a topic for future investigation.

In conclusion, our detailed large‐scale analysis reveals for the first time that squamates contain a variable source of postnatal proliferating progenitors in a pseudostratified RCJ at the peripheral retina. Strikingly, the RCJ shares with the CMZ a common pseudostratified nature, pattern of slow‐cycling cells and progenitor markers, and response to postnatal ocular growth, suggesting that it might be a functionally equivalent structure. Our new findings also highlight the remarkable variation in activity and location of proliferative peripheral retina structures among and within vertebrate species, indicating that the currently accepted general scenario of reduced CMZ activity across evolution is too simplistic.

## CONFLICT OF INTEREST

The authors declare no potential conflict of interest.

## AUTHOR CONTRIBUTIONS

J.E. and N.D.P. designed the experimental approach and selected the species sampling. J.E., L.S., and S.M. performed all experiments and 3D image segmentation. J.E., L.S., S.M., and N.D.P. collected and prepared most of the reptile embryos. J.E. and N.D.P. prepared the figures and wrote the paper, and L.S. and S.M. contributed in the form of discussion and critical comments. All authors approved the final version of the manuscript.

## Supporting information


**Supplementary Table 1** List of squamate species, measurements, and references used in the study. Species are classified by group and family names.

## Data Availability

3D CT data and ISH probes are available from the corresponding author upon reasonable request. All other data generated or analyzed during this study are included in the manuscript and supplementary file.
