# Supplementary material for: Variations in the proliferative activity of the peripheral retina correlate with postnatal ocular growth in squamate reptiles
Source: J Comp Neurol. 2019 Mar 28;527(14):2356–70. doi: 10.1002/cne.24677 (PMC6766921; doi:10.1002/cne.24677)
Supplement: Supplementary file 1 — Supplementary Table 1 List of squamate species, measurements, and references used in the study. Species are classified by group and family names. [file CNE-527-2356-s001.pdf]

Supplementary Table 1. List of squamate species, measurements, and references used in the study.

Groups	Families	Species	Eye axial length (mm)	Head length (mm)	References	Average number of proliferating cells per RCJ section
Anguimorpha	Anguidae	*Gerrhontus weighmanii*	6.00	24.20	Hall 2009**	
Anguimorpha	Anguidae	*Ophisaurus apodus*	3.00	17.50	This work	0.05
Anguimorpha	Helodermatidae	*Heloderma sp*	8.23	57.68	Hall 2009**	
Anguimorpha	Varanidae	*Varanus indicus*	8.05	54.01	Hall 2008*	
Gekkota	Diplodactylidae	*Naultinus elegans*	5.02	16.89	Hall 2009**	
Gekkota	Eublepharidae	*Coleonyx elegans*	4.62	20.46	Hall 2009**	
Gekkota	Eublepharidae	*Coleonyx variegatus*	2.59	12.02	Hall 2009**	
Gekkota	Eublepharidae	*Eublepharis hardwickii*	6.71	24.71	Hall 2009**	
Gekkota	Eublepharidae	*Eublepharis macularis*	5.00	26.00	This work	0.48
Gekkota	Gekkonidae	*Cyrtodactylus louisiadensis*	11.59	34.45	Hall 2009**	
Gekkota	Gekkonidae	*Gehyra variegata*	3.33	11.42	Hall 2009**	
Gekkota	Gekkonidae	*Gekko gecko*	10.00	53.00	This work	2.07
Gekkota	Gekkonidae	*Gekko swinhonis*	3.63	13.75	Hall 2009**	
Gekkota	Gekkonidae	*Hemidactylus mabouia*	4.36	15.42	Hall 2009**	
Gekkota	Gekkonidae	*Lepidodactylus lugubris*	3.26	9.04	Hall 2009**	
Gekkota	Gekkonidae	*Lygodactylus picturatus*	3.26	8.87	Hall 2009**	0.13
Gekkota	Gekkonidae	*Narudasia festiva*	2.23	8.59	Hall 2009**	
Gekkota	Gekkonidae	*Phelsuma astriata*	3.81	10.46	Hall 2009**	
Gekkota	Gekkonidae	*Phelsuma laticaudata*	3.59	13.26	Hall 2009**	
Gekkota	Gekkonidae	*Phelsuma madagascariensis*	4.20	20.23	This work	0.35
Gekkota	Phyllodactylidae	*Phyllodactylus reissii*	4.60	17.15	Hall 2009**	
Gekkota	Phyllodactylidae	*Ptyodactylus hasselquistii*	4.13	16.13	Hall 2009**	

Gekkota	Phyllodactylidae	*Tarentola mauritanica*	4.12	12.40	Hall 2009**	
Gekkota	Sphaerodactylidae	*Gonatodes ocellatus*	3.43	10.62	Hall 2009**	
Gekkota	Sphaerodactylidae	*Gonatodes vittatus*	2.83	8.53	Hall 2009**	
Gekkota	Sphaerodactylidae	*Pristurus carteri*	5.08	15.19	Hall 2009**	
Gekkota	Sphaerodactylidae	*Quedenfeldtia moerens*	4.03	11.73	Hall 2009**	
Gekkota	Sphaerodactylidae	*Sphaerodactylus anthracinus*	2.49	8.90	Hall 2009**	
Iguania	Agamidae	*Agama agama*	6.00	22.00	This work	0.50
Iguania	Agamidae	*Calotes mystaceus*	9.51	22.99	Hall 2009**	
Iguania	Agamidae	*Calotes versicolor*	7.61	25.21	Hall 2009**	
Iguania	Agamidae	*Draco melanopogon*	5.74	12.68	Hall 2009**	
Iguania	Agamidae	*Gonocephalus grandis*	7.86	31.34	Hall 2009**	
Iguania	Agamidae	*Gowidon longirostris*	5.76	23.93	Hall 2009**	
Iguania	Agamidae	*Leiolepis belliana*	8.98	27.70	Hall 2009**	
Iguania	Agamidae	*Physignathus cocincinus*	7.35	25.13	This work	1.41
Iguania	Agamidae	*Pogona vitticeps*	6.00	18.00	This work	3.05
Iguania	Agamidae	*Uromastyx hardwickii*	7.26	26.40	Hall 2009**	
Iguania	Chameleonidae	*Bradypodion pumilum*	3.62	15.33	This work	
Iguania	Chameleonidae	*Brookesia superciliaris*	3.45	8.77	Hall 2009**	
Iguania	Chameleonidae	*Chamaeleo africanus*	7.73	28.51	Hall 2009**	
Iguania	Chameleonidae	*Chamaeleo bitaeniatus*	5.46	16.57	Hall 2008*	
Iguania	Chameleonidae	*Chamaeleo calyptratus*	6.75	28.00	This work	1.80
Iguania	Chameleonidae	*Chamaeleo chamaeleon*	7.39	24.21	Hall 2009**	
Iguania	Chameleonidae	*Chamaeleo hoehnelli*	5.58	24.85	Hall 2009**	
Iguania	Chameleonidae	*Furcifer lateralis*	6.72	20.50	Hall 2009**	
Iguania	Chameleonidae	*Furcifer verrucosus*	6.39	25.33	Hall 2009**	

Iguania	Chameleonidae	*Rieppeleon brevicaudatus*	3.96	13.45	This work	0.67
Iguania	Chameleonidae	*Trioceros jacksonii*	8.16	27.58	This work	0.44
Iguania	Corytophanidae	*Basiliscus basiliscus*	7.80	35.76	Hall 2008*	
Iguania	Corytophanidae	*Basiliscus vittatus*	5.39	25.72	This work	1.56
Iguania	Crotaphytidae	*Crotaphytus bicinctores*	3.32	21.25	Hall 2009**	
Iguania	Dactyloidae	*Anolis carolinensis*	4.40	21.00	This work	0.27
Iguania	Dactyloidae	*Anolis trinitatis*	4.00	35.02	Hall 2008*	
Iguania	Iguanidae	*Amblyrhynchus cristatus*	7.04	31.23	Hall 2009**	
Iguania	Iguanidae	*Ctenosaura hemilopha*	6.10	22.96	Hall 2009**	
Iguania	Iguanidae	*Ctenosaura similis*	7.84	29.85	Hall 2009**	
Iguania	Iguanidae	*Dipsosaurus dorsalis*	6.84	29.11	Hall 2009**	
Iguania	Iguanidae	*Iguana iguana*	8.65	28.32	Hall 2009**	1.44
Iguania	Iguanidae	*Sauromalus ater*	7.88	30.80	Hall 2009**	
Iguania	Iguanidae	*Sauromalus obesus*	7.90	40.20	Hall 2008*	
Iguania	Leiocephalidae	*Leiocephalus carinatus*	6.03	22.62	Hall 2009**	
Iguania	Leiocephalidae	*Leiocephalus schreibersii*	5.31	21.30	This work	0.25
Iguania	Opluridae	*Oplurus cyclurus*	6.27	26.55	This work	
Iguania	Phrynosomatidae	*Callisaurus draconoides*	4.39	14.33	Hall 2009**	
Iguania	Phrynosomatidae	*Phrynosoma blainvillii*	6.55	29.35	Hall 2008*	
Iguania	Phrynosomatidae	*Sceloporus horridus*	6.28	22.32	Hall 2009**	
Iguania	Phrynosomatidae	*Sceloporus magister*	7.18	28.13	Hall 2009**	
Iguania	Phrynosomatidae	*Sceloporus oberon*	5.37	21.10	Hall 2009**	
Iguania	Phrynosomatidae	*Sceloporus occidentalis*	4.80	18.99	Hall 2008*	
Iguania	Phrynosomatidae	*Uma exsul*	5.05	15.72	Hall 2009**	
Iguania	Tropiduridae	*Tropidurus hispidus*	6.76	28.55	Hall 2009**	

Iguania	Tropiduridae	*Uranoscodon superciliosus*	8.79	34.96	Hall 2009**	
Lacertoidea	Lacertidae	*Acanthodactylus boskianus*	3.33	14.06	Hall 2009**	
Lacertoidea	Lacertidae	*Acanthodactylus cantoris*	4.12	16.33	Hall 2009**	
Lacertoidea	Lacertidae	*Acanthodactylus longipes*	2.60	10.23	Hall 2009**	
Lacertoidea	Lacertidae	*Acanthodactylus pardalis*	2.85	13.85	Hall 2009**	
Lacertoidea	Lacertidae	*Aporosaura anchietae*	3.90	13.56	Hall 2009**	
Lacertoidea	Lacertidae	*Eremias persica*	4.06	21.66	Hall 2009**	
Lacertoidea	Lacertidae	*Gallotia atlantica*	3.51	19.65	Hall 2009**	
Lacertoidea	Lacertidae	*Gallotia galloti*	4.76	27.72	Hall 2009**	
Lacertoidea	Lacertidae	*Heliobolus lugubris*	2.89	11.64	Hall 2009**	
Lacertoidea	Lacertidae	*Ichnotropis squamulosa*	3.90	16.63	Hall 2009**	
Lacertoidea	Lacertidae	*Lacerta agilis*	3.99	17.20	Hall 2009**	
Lacertoidea	Lacertidae	*Lacerta lepida*	4.63	19.42	Hall 2008*	
Lacertoidea	Lacertidae	*Lacerta viridis*	4.69	23.59	Hall 2009**	
Lacertoidea	Lacertidae	*Meroles knoxii*	3.42	14.09	Hall 2009**	
Lacertoidea	Lacertidae	*Mesalina guttulata*	2.99	11.05	Hall 2009**	
Lacertoidea	Lacertidae	*Ophisops sp.*	2.94	11.17	Hall 2009**	
Lacertoidea	Lacertidae	*Podarcis muralis*	3.24	16.94	Hall 2009**	
Lacertoidea	Lacertidae	*Takydromus septentrionalis*	3.04	16.05	Hall 2009**	
Lacertoidea	Lacertidae	*Takydromus sexlineatus*	2.50	13.00	This work	0.00
Lacertoidea	Teiidae	*Ameiva ameiva*	4.43	25.53	This work	
Lacertoidea	Teiidae	*Dracaena guianensis*	12.01	62.63	Hall 2009**	
Lacertoidea	Teiidae	*Tupinambis nigropunctatus*	9.00	31.69	Hall 2009**	
Scincoidea	Cordylidae	*Chamaesaura macrolepis*	2.93	15.43	Hall 2009**	
Scincoidea	Cordylidae	*Cordylus cordylus*	4.85	24.62	Hall 2009**	

Scincoidea	Cordylidae	*Cordylus niger*	4.19	22.50	Hall 2009**	
Scincoidea	Cordylidae	*Cordylus polyzonus*	5.33	26.93	Hall 2009**	
Scincoidea	Cordylidae	*Platysaurus guttatus*	5.10	25.16	Hall 2008*	
Scincoidea	Cordylidae	*Platysaurus intermedius*	3.78	19.32	Hall 2009**	
Scincoidea	Gerrhosauridae	*Gerrhosaurus major*	6.01	28.00	Hall 2009**	
Scincoidea	Gerrhosauridae	*Gerrhosaurus nigrolineatus*	5.07	22.25	Hall 2009**	
Scincoidea	Scincidae	*Chalcides ocellatus*	3.17	43.16	Hall 2008*	
Scincoidea	Scincidae	*Dasia olivacea*	4.80	22.00	This work	1.23
Scincoidea	Scincidae	*Egernia frerei*	6.44	35.41	Hall 2009**	
Scincoidea	Scincidae	*Egernia whitei*	3.44	16.54	Hall 2009**	
Scincoidea	Scincidae	*Eugongylus rufescens*	4.32	21.14	Hall 2009**	
Scincoidea	Scincidae	*Eumeces schneideri*	4.10	80.89	Hall 2008*	
Scincoidea	Scincidae	*Eumeces skiltonianus*	3.00	17.76	Hall 2008*	
Scincoidea	Xantusiidae	*Lepidophyma gaigeae*	2.69	12.94	Hall 2009**	
Scincoidea	Scincidae	*Lepidothyris fernandi*	4.32	22.97	This work	0.07
Scincoidea	Scincidae	*Mabuya perrotetii*	5.40	21.80	Hall 2009**	
Scincoidea	Scincidae	*Melanoceps loveridgei*	1.14	9.94	This work	0.38
Scincoidea	Scincidae	*Scincus mitranus*	5.15	18.70	Hall 2009**	
Scincoidea	Scincidae	*Scincus scincus*	4.10	38.23	Hall 2008*	
Scincoidea	Scincidae	*Sphenomorphus nigricauda*	2.54	13.27	Hall 2009**	
Scincoidea	Scincidae	*Tiliqua gigas*	8.01	55.63	Hall 2009**	
Scincoidea	Xantusiidae	*Xantusia henshawi*	2.97	13.28	Hall 2009**	
Scincoidea	Xantusiidae	*Xantusia riversiana*	4.91	21.13	Hall 2009**	
Scincoidea	Xantusiidae	*Xantusia vigilis*	1.72	8.36	Hall 2009**	
Serpentes	Boidae	*Eryx jaculus*	1.52	12.55	This work	0.22

Serpentes	Colubridae	*Chrysopelea ornata*	4.85	21.44	This work	0.00
Serpentes	Colubridae	*Dasypeltis gansi*	2.39	9.83	This work	0.00
Serpentes	Colubridae	*Pantherophis guttatus*	3.00	18.00	This work	0.04
Serpentes	Lamprophiidae	*Boaedon fuliginosus*	3.00	14.00	This work	
Serpentes	Pythonidae	*Python regius*	4.27	25.98	This work	0.50

*Hall, M.I. (2008). *Zoology* , **111**, 62-75.

Hall, M.I. (2009). *Anatomical Records* **292, 798-812
